# Dual burden of chronic physical conditions and mental disorders: Findings from the Saudi National Mental Health Survey

**DOI:** 10.3389/fpubh.2023.1238326

**Published:** 2023-11-28

**Authors:** Yasmin Altwaijri, Abdulhameed Al-Habeeb, Abdullah Al-Subaie, Ronny Bruffaerts, Lisa Bilal, Sanaa Hyder, Mohammad Talal Naseem, Abdullah J. Alghanim

**Affiliations:** ^1^Biostatistics, Epidemiology and Scientific Computing Department, King Faisal Specialist Hospital and Research Centre, Riyadh, Saudi Arabia; ^2^Research Department, King Salman Center for Disability Research, Riyadh, Saudi Arabia; ^3^SABIC Psychological Health Research & Applications Chair (SPHRAC), College of Medicine, King Saud University, Riyadh, Saudi Arabia; ^4^National Center for Mental Health Promotion, Ministry of Health, Riyadh, Saudi Arabia; ^5^Department of Psychiatry, Edrak Medical Center, Riyadh, Saudi Arabia; ^6^Universitair Psychiatrisch Centrum - Katholieke Universiteit Leuven (UPC-KUL), Campus Gasthuisberg, Leuven, Belgium; ^7^Psychiatry Department, King Fahd University Hospital, Khobar, Saudi Arabia

**Keywords:** comorbidity, chronic physical conditions, mental disorders, Saudi Arabia, disability

## Abstract

**Introduction:**

Comorbidities of mental disorders and chronic physical conditions are a common medical burden reported among Western countries. National estimates of such comorbidities among the general population of Arab countries like Saudi Arabia are unknown. This study examined the prevalence of lifetime chronic physical conditions among the Saudi general population with DSM-IV 12-month mental disorders, and the associations with disability in the Kingdom of Saudi Arabia (KSA).

**Methods:**

The Saudi National Mental Health Survey, a cross-sectional household study – part of the World Mental Health (WMH) Survey Consortium – was conducted between 2013–2016 in the KSA, with 4,001 Saudi citizens aged 15–65 (response rate 61%). The World Health Organization Composite International Diagnostic Interview 3.0 was used to assess prevalence of lifetime chronic physical conditions and 12-month mental disorders; disability was measured in terms of days out of role.

**Results:**

The prevalence of any comorbid 12-month mental disorder among those with chronic physical conditions was 24%. Major depressive disorder, social phobia, and adult separation anxiety disorder were the most common comorbid mental disorders across all chronic physical conditions. Gender, education, income, urbanicity, region, and employment were associated with the presence of any chronic physical condition. Respondents with mental / physical comorbidities had 2.97 days out of role (on average) in the last 30 days.

**Conclusion:**

Comorbidities of mental disorders and chronic physical conditions are common among Saudis. National efforts are needed to increase awareness of such comorbidities among the general population, and develop prevention and treatment services tailored to the needs of individuals at-risk for comorbidities.

## 1 Introduction

Medical comorbidity is a substantial and common societal burden, posing important challenges in terms of proper identification, prevention, and management strategies on the personal, clinical, and societal level. Comorbidity is usually defined as the presence of one or more additional diseases in a person diagnosed with an index-disease, i.e., a physical or mental condition (1, 2). However, such a co-presence does not imply that one disease is more central or severe than the other (3). Comorbidities between mental and physical disorders are commonly prevalent among general populations globally, regardless of the chronological order of onset, or the causal pathway linking the conditions (2, 4). For instance, individuals with chronic physical conditions are reported to have a higher prevalence of mental disorders than those without chronic physical conditions (2); the physical conditions were also more chronic when a mental disorder was present (5). The New Zealand Mental Health Survey found mental disorders among nearly 68% of individuals with medical conditions, particularly comorbidities related to high rates of depression and anxiety (6). The National Comorbidity Survey Replication in the United States found physical disorders among more than 68% of adults with a mental disorder and mental disorders among 29% of adults with a physical disorder (7). A meta-analysis reported that mental/physical comorbidities ranged from 19.0 to 68.1% in developing countries (8). Other global studies suggest an association between chronic pain conditions and mental disorders, including mood, anxiety, somatoform, substance use, and personality disorders (9–15). Such comorbidities have been largely linked to functional disability and significant loss of quality of life (10, 13).

The Arab world reports similar findings on mental/physical comorbidities (16). According to a study conducted in Aleppo, Syria, depression was higher in people with stroke and heart disease (17). In the context of the Kingdom of Saudi Arabia (KSA), up to 30% of patients with colorectal cancer meet criteria for major depressive disorder (18). Other Saudi studies suggest a strong association between depression and cardiovascular disease (19), high rates of anxiety and depression among children and adolescents with chronic pain conditions (20), with 71% of those with a pain condition related to a disease — such as cancer and osteoarthritis — meeting criteria of depression (21).

Specific sociodemographic risk factors for mental/physical comorbidity include: higher age, female gender, and lower education (22). Those with pain conditions in the KSA, who are financially discontent, are three times more likely to have depression compared to those who are financially content (21). Compared to other age groups, Saudis aged 40–59 with chronic pain have an increased risk of depression (21). Mental disorders and chronic physical conditions are associated with higher levels of disability and role impairment among Saudis (23, 24): yet only a small percentage receive or seek treatment (25). The effects of comorbid mental disorders are reported to lead to higher treatment costs, functional impairment, and prolonged duration of treatment (23, 26). It is important to note that there are several possible reasons for such comorbidities to exist: (1) the mental disorder causes the chronic physical condition (or vice versa), (2) the conditions influence and mutually maintain each other in some way, (3) a third factor (e.g., a sociodemographic characteristic) increases the risk of developing both mental disorders and chronic physical conditions, or (4) the conditions occur independently, by a mechanism that is unrelated to the other (27). These outlined findings emphasize the critical need for evidence based-studies aimed at understanding the burden of comorbidities, in order to develop policy frameworks for interventions to prevent further comorbidity development.

Although informative, basic epidemiological findings on mental/physical comorbidity in the KSA general population are lacking. Overall, previous Saudi studies have focused on such comorbidity patterns between selected chronic physical conditions and mental disorders (22, 28–30), and this among specific populations (31). To the best of our knowledge, there are no published studies that have used rigorous field methods to examine comorbidities of broad chronic physical conditions and mental disorders, and the correlates of such comorbidities among the general population in the KSA.

This study uses data collected by the Saudi National Mental Health Survey (SNMHS), a national epidemiological survey conducted in the KSA, as part of the World Mental Health (WMH) Survey Initiative (32). Under the WMH Consortium (33), all participating countries conduct general population surveys using the World Health Organization (WHO) WMH Composite International Diagnostic Interview 3.0 (CIDI) to retrospectively assess mental disorders, based on the *Diagnostic and Statistical Manual of Mental Disorders* IV (DSM-IV). The present study is one of the first Arab studies under the WMH Consortium to investigate the relationship between mental disorders and chronic conditions. The aims of the study are to examine (i) the prevalence of DSM-IV 12-month mental disorders among Saudis with lifetime chronic physical conditions, (ii) the sociodemographic correlates of chronic physical conditions, (iii) the interaction of 12-month mental disorders with sociodemographic correlates of chronic physical conditions, and (iv) the associations between such comorbidities and disability among Saudis in the KSA. National estimates on such comorbidities are needed to inform mental health stakeholders, and policymakers involved in improving the quality of life of the affected individuals.

## 2 Materials and methods

### 2.1 Sample

The sample was obtained from the SNMHS. As described previously, (34, 35) the SNMHS was based on a multi-stage clustered probability sample of Arabic-speaking household citizens aged between 15 and 65 years old, living in urban and rural areas of the KSA. Over 28 months between 2014 and 2016, trained lay interviewers conducted face-to-face interviews, as per the general methods of the WMH survey initiative. The response rate for a total sample of 4,004 participants was 61%. The interviews were conducted in two parts in an effort to reduce respondent burden and control study costs, thereby enhancing the data collection process and enabling a comprehensive assessment of research objectives. Part I included a core diagnostic assessment that was given to all respondents. Part II included questions about risk factors, consequences, other correlates, and assessments of additional disorders. Part II was given to all Part I respondents who had any core disorder, as well as a 25% probability sub-sample of other Part I respondents (n = 1981). Prior to the interview, the interviewers obtained written informed consent. This study was performed in line with the principles of the Declaration of Helsinki. Field procedures and consent were approved by the Institutional Review Board committee at the King Faisal Specialist Hospital & Research Centre, Riyadh, Saudi Arabia. The characteristics of the sub-sample with chronic physical conditions are presented in Table 1.

**Table 1:** Prevalence of chronic physical conditions in the total sample, and the characteristics of the sub-sample.

	*N*	%	SE
**Any physical condition**	1,538	69.07	1.99
Medical condition	476	19.67	1.41
Pain condition	1,479	65.62	2.02
**Any mental disorders** (*N* = 1,538)			
Yes	545	24.00	1.56
No	993	76.00	1.56
**Gender**			
Female	967	57.74	2.23
Male	571	42.26	2.23
**Age**			
15–22	374	25.45	1.96
23–30	357	22.90	1.87
31–49	609	36.49	2.15
50+	198	15.15	1.83
**Education**			
Low	266	19.26	1.88
Low Average	211	13.56	1.49
High Average	515	31.16	2.08
High	546	36.02	2.17
**Income**			
Low	648	44.76	2.31
Low Average	152	8.65	1.14
High Average	231	15.54	1.51
High	507	31.05	1.99
**Marital status**			
Married	901	51.80	2.27
Separated/Divorced/Widowed	117	7.87	1.28
Never Married	520	40.33	2.25
**Urban/Rural**			
Urban	1,369	84.99	1.99
Rural	169	15.01	1.99
**Region**			
Central	536	32.97	2.04
Eastern	217	19.59	1.92
Northern	152	3.90	0.50
Southern	167	11.26	1.27
Western	466	32.28	2.25
**Employment**			
Employed	473	31.10	2.03
Self-Employed	29	3.09	0.93
Unemployed	120	7.07	1.05
Retired	53	4.60	1.11
Homemaker	477	27.13	2.08
Student	361	24.65	1.96
Other ^**Ψ**^	25	2.36	0.60

Ψ Other includes disabled, maternity leave, sick leave, do not know, refused.

Part II weights were used.

### 2.2 Measures

#### 2.2.1 Mental disorders

The mental disorder diagnoses were based on the WHO-WMH CIDI 3.0 – a fully structured lay administered interview that generates diagnoses according to the criteria of DSM-IV (36). The computerized version of CIDI 3.0 was translated into Arabic and adapted to suit the local culture, then validated in the Arabic-speaking local population (34, 37, 38). The clinical reappraisal study of the Saudi CIDI showed valid and conservative diagnoses of common mental disorders in the Saudi population (39).

Twelve-month disorders assessed by the instrument criteria were used, and classified as follows: anxiety disorders (panic disorder, generalized anxiety disorder, agoraphobia without panic disorder, social phobia, posttraumatic stress disorder, obsessive-compulsive disorder, separation anxiety disorder), mood disorders (major depressive disorder, bipolar disorder I or II), impulse control disorders (conduct disorder, attention-deficit/hyperactivity disorder, intermittent explosive disorder), substance use disorders (alcohol and drug abuse and dependence) and eating disorders (anorexia, binge eating disorder, bulimia). The DSM-IV organic exclusion rules and diagnostic hierarchy rules were applied to the diagnoses as described elsewhere (24).

#### 2.2.2 Chronic physical conditions

Lifetime chronic conditions were assessed as part of the adapted CIDI 3.0, and were grouped into the following categories in this study: any medical condition (heart disease, high blood pressure, asthma, other chronic lung diseases [like chronic obstructive pulmonary disease, tuberculosis or emphysema], cancer; any pain condition (arthritis, chronic back or neck pain, frequent or severe headaches, other chronic pain); and any physical condition (any condition reported in either the medical or pain category).

#### 2.2.3 Sociodemographic correlates

Sociodemographic correlates included age (15–24 years, 25–34 years, 35–49 years, and 50 and above years), gender, completed years of education (0–6 years, i.e., low; 7–9 years, i.e., low-average; 10–15 years, i.e., high-average; and 16 years or more, i.e., high), and marital status (married, separated or widowed or divorced, and never married). Family income was defined as the household income divided by the number of people in the household. A case was assigned a category on a scale based on the *per capita* income of the respondent’s household divided by the median income for the country, where it was categorized as low if it was less than half the median, low-average if half the whole median, high-average if up to twice the median, and high if greater than twice the median. Employment included those who are employed, self-employed, unemployed, retired, a homemaker, a student, and others (such as disabled, maternity leave, sick leave, refused to answer, do not know). Urbanicity (rural, and urban) and region (Central [Riyadh, Al Qaseem, Hail], Eastern [Eastern Province], Northern [Northern Frontier, Al-Jouf, Tabouk], Southern [Aseer, Al-Baha], and Western [Makkah, Al-Madinah]) (35) were extracted from the sample frame (2010 Census) that was provided by the General Authority for Statistics in Saudi Arabia.

#### 2.2.4 Disability

Assessment of disability was similar to previous WMH studies (23, 40). Disability was measured in terms of ‘days out of role’ in the last 30 days, using the Work Loss Days (WLD) index of the World Health Organization Disability Assessment Schedule (WHODAS-II) (41). The WLD index assesses both days with full disability (i.e., in which the respondent is totally unable to perform daily tasks as usual (42), and days with partial disability (43). Full disability was estimated by asking respondents the number of days in the 30 days before interview (that is, beginning yesterday and going back 30 days) they were totally unable to work or carry out your normal activities because of problems with either their physical health, mental health, or use of alcohol or drugs. Partial disability was defined as the number of days in which respondents (a) had to cut down on what they did, assessed by the following item ‘How many days out of the past 30 were you able to work and carry out your normal activities, but had to cut down on what you did or not get as much done as usual because of problems with either your physical health, your mental health, or your use of alcohol or drugs?’; (b) had to cut back on the quality of what they did, assessed by the following item: ‘How many days out of the past 30 did you cut back on the quality of your work or how carefully you worked because of problems with either your physical health, your mental health, or your use of alcohol or drugs?’; and (c) experienced extreme effort to perform as usual, assessed by the following item: ‘How many days out of the past 30 did it take an extreme effort to perform up to your usual level at work or at your other normal daily activities because of problems with either your physical health, your mental health, or your use of alcohol or drugs?’. Using these four questions, a weighted sum of activity limitation days in the prior month was estimated. The following terms were added together: (1) The number of days totally unable to carry out normal activities in the prior month; (2) One-half the number of days of reduced activities; (3) One-half the number of days of reduced quality or care in work activities; and (4) One-quarter the number of days requiring extreme effort to perform at one’s usual level. If this sum exceeded 30, it was recoded to equal 30 so that the sum had a range from 0 to 30. The sum was then divided by 30 and multiplied by 100 so that the resulting WLD index score also ranged from 0 to 100.

### 2.3 Analysis

Sample weights were used to adjust for differential probabilities of selection in between-household and within-household as well as sample and population distrubutions. In brief, Part I weights were used for core-section disorders, while Part II weights were used for noncore-section disorders. Further detail on weighting procedures can be found elsewhere (35). For chronic physical conditions, respondents who reported any medical condition, and any pain condition were counted once each to calculate the total frequencies for both categories; and counted only once for the total frequency of the third category, i.e., any physical condition. Similarly, for all classes of mental disorders (e.g., any anxiety disorders), respondents who reported comorbid mental disorders from other classes of mental disorders (e.g., any mood disorders) were counted once each to calculate total frequencies. Prevalence of mental disorders with each comorbid chronic condition were obtained using crosstabulations with appropriate weights (35) for each disorder. Differences across chronic physical conditions and mental disorders classes were considered at *p*-value threshold of <0.05, using cross tabulations with the Wald chi-square test. The cross tabulations were done using the PROC SURVEYFREQ procedure in SAS 9.2 (SAS Institute Inc., Cary, NC, USA). Multiple logistic regression models were created to investigate associations between the chronic physical conditions, mental disorders, and sociodemographic correlates, as well as for the interaction between comorbidities and sociodemographic correlates. All logistic regression models were created using the PROC LOGISTIC procedure, and reported using odds ratios with 95% confidence intervals, with Wald chi-square tests with *p*-value threshold of <0.05. The WLD means were also calculated with 95% confidence intervals using the PROC MEANS procedure.

## 3 Results

The prevalence of any lifetime chronic physical condition among Saudis was 69.1%; and the prevalence of any 12-month comorbid mental disorder among those with chronic physical conditions was 24% (Table 1). The majority among those who reported chronic physical conditions were female, aged 31–49, with high education and low income, married, living in an urban area, in the Central or Western region, or employed.

### 3.1 Mental disorders among respondents with physical conditions

Major depressive disorder (5.9%), social phobia (5.8%), and adult separation anxiety disorder (5.8%) were the most common comorbid mental disorders among Saudis with any medical condition (Table 2). By comparison, social phobia (5.4%) and major depressive disorder (4.6%) were the most prevalent comorbid mental disorders among respondents with any pain condition. These disorders were also the most common comorbid mental disorders among Saudis with any physical condition (with prevalence estimates of 5.2 and 4.8%, respectively).

**Table 2:** Twelve-month Prevalence of Mental Disorders among Saudis with Chronic Physical Conditions.

Disorders	Any medical condition		Any pain condition		Any physical condition	
	*N*	%	*N*	%	*N*	%
**Anxiety disorders**						
Panic disorder^1^	10	0.8	42	1.7	42	1.6
Generalized anxiety disorder^1^	13	2.1	30	1.2	31	1.3
Social phobia^1^	38	5.8	113	5.4	113	5.2
Agoraphobia with/without panic^1^	21	1.7	67	2.5	68	2.4
Post-traumatic stress disorder^2^	19	2.3	56	2.5	56	2.4
Adult separation anxiety disorder^2^	28	5.8	90	4.5	90	4.3
Obsessive-compulsive disorder^2^	18	2.4	56	2.3	56	2.2
**Mood disorders**						
Major depressive disorder^1^	41	5.9	138	4.6	140	4.8
Bipolar I and/or II^1^	30	5	77	3.5	77	3.3
**Impulse control disorders**						
Conduct disorder^2^	5	1.1	7	0.4	7	0.3
Attention deficit hyperactivity disorder^2^	24	3.2	78	3.5	79	3.3
Intermittent explosive disorder^2^	25	5.7	56	2.8	58	3
**Substance use disorders**						
Alcohol abuse^2^	2	0.1	4	0.1	4	0.1
Alcohol dependence^2^	0	-	5	0.3	5	0.3
Drug abuse^2^	14	2.2	32	1.5	33	1.4
Drug dependence^2^	7	0.9	13	0.6	13	0.6
**Eating disorders**						
Anorexia^2^	0	-	0	-	0	-
Binge eating disorder^2^	20	4.5	40	2.5	42	2.4
Bulimia^2^	14	1.8	31	1.2	31	1.1
**More than one disorder**^**2**^	40	8.15	87	3.91	88	4.05

1 Part I sample, prevalence calculated using part I weights.

2 Part II sample, prevalence calculated using part II weights.

*N* = 4,004.

With regard to impulse control disorders, high rates of attention deficit disorder and intermittent explosive disorder were found among Saudis belonging to all three categories of chronic physical conditions. Binge eating disorder was particularly high (4.5%) among Saudis with any medical condition. Relative to other disorders, substance use disorders such as alcohol abuse (0.1%) were not as prevalent as anxiety disorders and mood disorders among Saudis with chronic physical conditions. Anxiety disorders and mood disorders were the most common comorbid mental disorders across all categories of chronic physical conditions (Table 3). Eating disorders (39.25%) and impulse control disorders (33.57%) were only prevalent and comorbid among Saudis with any medical condition.

**Table 3:** Prevalence of comorbidities stratified by classes of 12-month mental disorders.

Chronic condition	Anxiety disorders^2(N = 367)^				Mood disorders^1(N = 244)^				Eating disorders^2(N = 91)^				Impulse Control disorders^2(N = 158)^					Substance Use disorders^2(N = 53)^				More than one disorder^2(N = 214)^		
	N	%	Wald χ2	Pr > F	N	%	Wald χ2	Pr > F	N	%	Wald χ2	Pr > F	N	%	Wald χ2	Pr > F	N	%	Wald χ2	Pr > F	N	%	Wald χ2	Pr > F
**Any medical condition**	101	28.31	6.34	0.01	71	34.43	6.59	0.01	34	39.25	5.87	0.02	49	33.57	4.79	0.03	18	27.66	0.62	0.43	70	38.66	10.86	0.001
**Any pain condition**	318	84.35	27.95	<0.0001	215	80.16	7.35	0.01	71	76.17	2.09	0.15	126	72.63	1.39	0.24	44	76.05	0.89	0.35	186	80.56	8.98	0.002
**Any physical condition**	320	84.81	20.54	<0.0001	217	83.76	8.90	0.00	73	76.78	1.15	0.28	129	77.17	2.21	0.14	45	76.45	0.44	0.51	187	83.89	10.35	0.001

^1^Part I sample; prevalence calculated using part I weights.

^2^Part II sample; prevalence calculated using part II weights.

The degree of freedom for all categories = 1.

### 3.2 Correlates of physical conditions

Females were three times more likely than males to report any pain condition, or any physical condition. Compared to older Saudis (50+), those in other age groups had lower risk of any medical condition. Those who had low-average, high average and high levels of education were less likely to report any medical condition (OR = 0.47, *p* = 0.0008; OR = 0.55, *p* = 0.0046; OR = 0.64, *p* = 0.0389), any pain condition (OR = 0.40, *p* < 0.0001; OR = 0.60, *p* = 0.0121; OR = 0.56, *p* = 0.0056), and any physical condition (OR = 0.37, *p* < 0.0001; OR = 0.50, *p* = 0.0010; OR = 0.55, *p* = 0.0064) compared to those who had low levels of education.

Saudis with low average and high average income had lower risk of any pain condition (OR = 0.69, *p* = 0.0624; OR = 0.70, *p* = 0.0279) and any physical condition (OR = 0.55, *p* = 0.0028; OR = 0.65, *p* = 0.0084) compared to those with high income. Being separated/divorced/widowed vs. married was associated with increased risk of any medical condition (OR = 1.74, *p* = 0.0103). Those living in rural areas compared to urban areas were less likely to report any pain condition (OR = 0.54, *p* < 0.0001), and any physical condition (OR = 0.60, *p* = 0.0003). Northern and Southern region vs. Central region of the KSA were associated with lower rates of any medical condition (OR = 0.40, *p* = 0.0087; OR = 0.54, *p* = 0.0073); Northern and Western region vs. Central region were associated with lower rates of any pain condition (OR = 0.35, *p* < 0.0001; OR = 0.69, *p* = 0.0039); and Northern, Southern and Western region vs. Central region were associated with lower rates of any physical condition (OR = 0.27, *p* < 0.0001; OR = 0.63, *p* = 0.0134; OR = 0.62, *p* = 0.0003).

Saudis, who were self-employed and students vs. employed had lower risk of any medical condition (OR = 0.23, *p* = 0.0077; OR = 0.48, *p* = 0.0160). Those who were retired were almost three times more likely than those who were employed to report any pain condition and any physical condition (OR = 2.89, *p* = 0.0025). Employment reports of other types (disabled, maternity leave, sick leave, do not know, refused, etc.) were associated with lower rates of any medical condition (OR = 0.32, *p* = 0.0178), any pain condition (OR = 0.45, *p* = 0.0164), and any physical condition (OR = 0.39, *p* = 0.0052).

Saudis without mental disorders had systematically lower risk of any medical condition (OR = 0.47, *p* < 0.0001), any pain condition (OR = 0.40, *p* < 0.0001), and any physical condition (OR = 0.44, *p* < 0.0001) compared to those who had a comorbid 12-month mental disorder (Table 4).

**Table 4:** Association of Chronic Physical Conditions and Correlates.

	Any Medical Condition			Any Pain Condition			Any Physical Condition		
	OR	(95% CI)	*p*-Value	OR	(95% CI)	*p*-Value	OR	(95% CI)	*p*-Value
**Any mental disorders** ^**2**^									
Yes	1.00			1.00			1.00		
No	0.47	(0.35–0.62)	<0.0001	0.40	(0.3–0.54)	<0.0001	0.44	(0.33–0.59)	<0.0001
Wald χ^2^	28.54			38.65			29.46		
**Gender**									
Female	1.29	(0.93–1.79)	0.1209	3.10	(2.37–4.04)	<0.0001	3.11	(2.36–4.1)	<0.0001
Male	1.00			1.00			1.00		
Wald χ^2^	2.41			69.16			65.27		
**Age**			0.0013			0.3819			0.8657
15–22	0.42	(0.2–0.87)	0.0186	0.68	(0.36–1.27)	0.2212	0.97	(0.51–1.85)	0.9279
23–30	0.35	(0.21–0.59)	<0.0001	0.72	(0.43–1.19)	0.1970	0.94	(0.56–1.59)	0.8222
31–49	0.63	(0.42–0.94)	0.0240	0.69	(0.45–1.05)	0.0837	0.86	(0.55–1.33)	0.4912
50+	1.00			1.00			1.00		
Wald χ^2^	15.70			3.06			0.73		
**Education**			0.0047			0.0003			<0.0001
Low	1.00			1.00			1.00		
Low Average	0.47	(0.3–0.73)	0.0008	0.40	(0.26–0.61)	<0.0001	0.37	(0.24–0.57)	<0.0001
High Average	0.55	(0.37–0.83)	0.0046	0.60	(0.4–0.89)	0.0121	0.50	(0.33–0.76)	0.0010
High	0.64	(0.42–0.98)	0.0389	0.56	(0.37–0.85)	0.0056	0.55	(0.36–0.85)	0.0064
Wald χ^2^	12.96			18.58			21.17		
**Income**			0.4012			0.0651			0.0076
Low	1.06	(0.77–1.46)	0.7229	0.74	(0.56–0.97)	0.0293	0.76	(0.57–1.01)	0.0163
Low Average	0.83	(0.52–1.35)	0.4605	0.69	(0.47–1.02)	0.0624	0.55	(0.37–0.82)	0.0028
High Average	0.80	(0.55–1.16)	0.2357	0.70	(0.52–0.96)	0.0279	0.65	(0.48–0.9)	0.0084
High	1.00			1.00			1.00		
Wald χ^2^	2.94			7.23			11.94		
**Marital status**			0.0371			0.7423			0.3547
Married	1.00			1.00			1.00		
Separated/Divorced/Widowed	1.74	(1.14–2.66)	0.0103	0.84	(0.51–1.38)	0.4830	1.27	(0.72–2.24)	0.4176
Never Married	1.11	(0.74–1.68)	0.6131	0.93	(0.66–1.32)	0.6817	0.82	(0.57–1.17)	0.2757
Wald χ^2^	6.59			0.60			2.07		
**Urban/Rural**									
Urban	1.00			1.00			1.00		
Rural	0.80	(0.56–1.14)	0.2236	0.54	(0.41–0.71)	<0.0001	0.60	(0.45–0.79)	0.0003
Wald χ^2^	1.48			18.93			12.94		
**Region**			0.0120			<0.0001			<0.0001
Central	1.00			1.00			1.00		
Eastern	0.84	(0.6–1.19)	0.3312	1.38	(1–1.9)	0.0527	1.19	(0.85–1.67)	0.3048
Northern	0.40	(0.21–0.8)	0.0087	0.35	(0.22–0.54)	<0.0001	0.27	(0.18–0.43)	<0.0001
Southern	0.54	(0.34–0.85)	0.0073	0.73	(0.51–1.04)	0.0784	0.63	(0.44–0.91)	0.0134
Western	0.77	(0.58–1.03)	0.0733	0.69	(0.53–0.89)	0.0039	0.62	(0.47–0.8)	0.0003
Wald χ^2^	12.86			41.02			48.57		
**Employment**			0.0053			0.0030			0.0029
Employed	1.00			1.00			1.00		
Self-Employed	0.23	(0.08–0.68)	0.0077	1.65	(0.92–2.94)	0.0911	1.49	(0.82–2.68)	0.1879
Unemployed	0.66	(0.37–1.17)	0.1560	1.24	(0.78–1.98)	0.3686	1.20	(0.74–1.94)	0.4531
Retired	1.43	(0.78–2.62)	0.2464	2.89	(1.45–5.74)	0.0025	2.75	(1.35–5.57)	0.0051
Homemaker	0.81	(0.53–1.23)	0.3160	1.09	(0.73–1.61)	0.6863	0.91	(0.6–1.37)	0.6531
Student	0.48	(0.27–0.87)	0.0160	1.04	(0.67–1.61)	0.8570	0.85	(0.55–1.33)	0.4847
Other ^**Ψ**^	0.32	(0.12–0.82)	0.0178	0.45	(0.23–0.86)	0.0164	0.39	(0.2–0.75)	0.0052
Wald χ^2^	18.41			19.78			19.91		

Ψ Other includes disabled, maternity leave, sick leave, do not know, and refused.

Part II Weights were used.

Mental disorders were found to interact with gender, age, income, and employment to exert an effect on the risk of any medical condition (Table 5). There were mental disorders interactions with all sociodemographic characteristics except for marital status for the category of any pain condition. Gender, education, income, urbanicity, region, and employment interacted with mental disorders to predict the risk of any physical condition. For instance, females with mental disorders were found to be at a considerably higher risk of developing physical conditions than males (*p* < 0.0001). Additionally, individuals with mental disorders who had high (*p* = 0.0009) and high-average (*p* = 0.0001) education levels had reduced risks of experiencing physical conditions. Individuals in rural areas were also at a notably lower risk of physical conditions (*p* < 0.0001). Furthermore, relative to those living in the Western region, individuals with mental disorders residing in the Central and Eastern regions (*p* = 0.0003 & 0.0003) were at a significantly higher risk for physical conditions, while those in the Northern region were at a lower risk (*p* = 0.0001) (Supplementary Table S1).

**Table 5:** Mental disorders interactions of sociodemographic correlates and chronic physical conditions.

Effect	Any medical condition			Any pain condition			Any physical condition		
	DF	Wald Chi-Square	Pr > ChiSq	DF	Wald Chi-Square	Pr > ChiSq	DF	Wald Chi-Square	Pr > ChiSq
Any mental disorders * gender	1	6.4862	0.0109	1	58.1913	<0.0001	1	51.9596	<0.0001
Any mental disorders * age	3	13.8958	0.0031	3	9.5056	0.0233	3	2.3549	0.5021
Any mental disorders * education	3	2.617	0.4545	3	19.7106	0.0002	3	20.4171	0.0001
Any mental disorders * income	3	7.6858	0.053	3	9.8179	0.0202	3	18.1043	0.0004
Any mental disorders * marital status	2	4.3148	0.1156	2	1.1484	0.5632	2	3.5203	0.172
Any mental disorders *urban/rural	1	1.8334	0.1757	1	28.1706	<0.0001	1	18.668	<0.0001
Any mental disorders * region	4	8.0999	0.088	4	39.6121	<0.0001	4	46.4706	<0.0001
Any mental disorders * employment	6	17.2664	0.0084	6	19.2934	0.0037	6	19.2506	0.0038

*Interaction between the two categories.

### 3.3 Mental/physical comorbidity and work loss days indices

We found meaningful associations between comorbidities and days out of role in the last 30 days (Table 6): respondents without mental disorders or chronic physical conditions had 0.27 days out of role (CI: 0.12–0.43, SE: 0.11) — on average — in the last 30 days. Those with 12-month mental disorders but without any physical conditions had 4.44 (CI: 2.73–6.15, SE: 0.86) days out of role. Saudis with chronic physical conditions but without any mental disorder had 1.18 (CI: 0.90–1.47, SE: 0.14) days out of role, and those with comorbidities of mental disorder and chronic physical condition had an average of 2.97 (CI: 2.45–3.49, SE: 0.26) days out of role in the last 30 days.

**Table 6:** Days out of role in the last 30 days for Saudis with 12-month mental disorders and lifetime chronic physical conditions.

Condition	Prevalence				Mean Days Out of Role in the last 30 days		
	*N*	%	SE	95% CI	Mean	SE	95% CI
Mental disorder only	94	3.62	0.61	(2.41–4.83)	4.44	0.86	(2.73–6.15)
Physical condition only	993	52.49	2	(48.56–56.42)	1.18	0.14	(0.90–1.47)
Both mental disorder and physical condition	545	16.58	1.09	(14.42–18.73)	2.97	0.26	(2.45–3.49)
No mental disorder or physical condition	349	27.31	1.97	(23.44–31.18)	0.27	0.11	(0.12–0.43)

Part II weights were used; *n* = 1981.

## 4 Discussion

This is one of the first Arab studies under the WMH Consortium to examine comorbidities of chronic physical conditions and mental disorders in the general population of a country from the Gulf Cooperation Council (GCC). We found that chronic physical conditions among Saudis were common. In addition, about one in four Saudis with chronic physical conditions reported comorbid mental disorders. Specifically social phobia, adult separation anxiety disorder, and major depressive disorder were common mental disorders across all chronic physical condition categories. Overall, anxiety disorders were commonly comorbid across all chronic physical conditions; a large proportion (84.34%) of Saudis with any pain condition had comorbid anxiety disorders, as well as any physical condition comorbid with anxiety disorders (84.81%). These results were similar to other WMH studies (4, 9, 11, 40, 44), as well as with Arab (16) and Saudi studies with chronic physical condition clinical patients (18, 21).

Also in line with global (4, 11, 13, 45, 46), Arab (16), and Saudi studies (21, 29, 47), Saudis without comorbid 12-month mental disorders had lower rates of chronic physical conditions. Also, our findings related to the burden of comorbidities, and disability in terms of days out of role were supported by the Global Burden of Disease Study in the KSA (48). These results highlight the role of such burdens on impairment and loss of productivity, thereby pointing to the importance of addressing the large impact of mental/physical comorbidities on quality of life in local healthcare reforms (23). Additionally, that substance use disorders were generally less associated with chronic physical conditions than has been previously reported (15) can be largely attributed to the ban on alcohol and drug consumption and the limited availability of substances in the country (49).

Older age, low education, being separated/divorced/widowed, living in the Central region of the KSA, and being employed were associated with increased risk of any medical condition. Mental disorders interacted with the sociodemographic correlates (excluding marital status) to predict the risk of chronic physical conditions categories among Saudis. Future research is warranted, given that this study did not examine the temporal relationships between the conditions, and causal mechanisms of such comorbidities. However, as previously mentioned, a number of relationship scenarios are possible between the conditions. It is possible that the mental disorder or chronic physical condition cause or influence one another in certain ways, or that certain sociodemographic factors increase vulnerability to either condition, or even that the conditions do not influence each other, but simply coincide (27).

Within the context of the KSA, the New Models of Care – outlined by the Ministry of Health for the Vision 2030, Health Sector Transformation Strategy (50) – specify chronic conditions as a prioritized system of care, and recognize that the mental health services in the country are underdeveloped, and need to be taken into account as the healthcare development progresses. Currently, the Saudi government ensures that ‘Comprehensive Health Guidance Initiative’ under the primary mental health program of the Ministry of Health, provides integrated mental health treatment – to some extent – in physical health treatment. The aims of this program include early detection of depression and anxiety among primary health centres’ outpatients, and provision of primary mental health services for prevention of chronic diseases (51). Previous Saudi reports also indicate that pharmacotherapy – specifically polypharmacy, i.e., use of multiple medication – is common among outpatients with medical conditions (52) and mental disorders (53). However it is unclear how effectively and explicitly comorbidities are targeted, or addressed in the healthcare sector, or across other sectors in the KSA generally. Saudi studies in the past has advocated for multidisciplinary approaches such as the biopsychosocial model to be considered by healthcare professionals, to address mental disorders among individuals with chronic physical conditions, especially in hospitals (21, 47). Furthermore, although the National Center for Mental Health Promotion provides accessible psychological education services to the public (54), tailored healthcare efforts, support services, and awareness campaigns explicitly addressing comorbidities of mental disorders and chronic physical conditions among the general population are needed.

The findings of this study must be interpreted in light of the following limitations. First, the assessment of comorbidities in this study may be different from that of a clinical population survey. Symptoms of mental disorders such as fatigue in depressive disorders may also be common for chronic illnesses; comorbidities in this study were assessed without applying any exclusionary criteria for entangled symptoms of physical and mental disorders (40). Also, psychotic disorders, such as schizophrenia, that did not fall within the scope of the SNMHS may also be commonly linked with medical comorbidities (55). Second, due to the stigma surrounding mental disorders and self-reporting of symptoms and chronic conditions, the prevalence of comorbidities in this study may be an underestimation. However, there is evidence to support that self-reports generally show good concordance with medical reports for chronic diseases (46, 56). Third, given the cross-sectional nature of this study, any indicated associations between comorbidities and correlates do not imply causation. Prospective research studies could investigate ages of onset of mental disorders and chronic physical conditions to understand the relationship between these disorders as well as determine the course of comorbid chronic conditions (9).

## 5 Conclusion

Using nationally representative data from the SNMHS, this study investigated the dual burden of mental disorders and chronic physical conditions and their association with sociodemographic correlates and disability. Our findings demonstrate that such comorbidities were common among the Saudi population, particularly mood and anxiety disorders. Mental/physical disorders were linked to a number of sociodemographic correlates as well as significant disability. Overall, the findings of this study provide a valuable point of comparison alongside other surveys from high-income countries under the WMH Consortium — the vast majority of which were conducted among Western populations. Our study also contributes to a growing body of Saudi literature, and provides national estimates related to comorbidities that were unavailable before. These estimates will inform social, mental and health policies across the country and the GCC, in terms of identifying vulnerable groups at risk for comorbidities, and developing prevention and treatment services tailored to their specific health needs.

## Data availability statement

The datasets presented in this article are not readily available because of restrictions in the informed consent language used to recruit respondents and WMH consortium agreements. Requests to access the datasets should be directed to the corresponding author at yasmint@kfshrc.edu.sa.

## Ethics statement

The studies involving humans were approved by Institutional Review Board committee at the King Faisal Specialist Hospital & Research Centre, Riyadh, Saudi Arabia. The studies were conducted in accordance with the local legislation and institutional requirements. Written informed consent for participation in this study was provided by the participants' legal guardians/next of kin.

## Author contributions

YA and LB: conceptualization and supervision. YA, LB, AA-S, and AA-H: project administration, investigation, funding acquisition, and resources. YA, LB, MN: methodology. MN: Formal analysis. LB, SH, and AA: writing - original draft preparation. YA, RB, LB, SH, and AA: writing - review and editing. All authors contributed to the article and approved the submitted version.
