# Supplementary material for: Dual burden of chronic physical conditions and mental disorders: Findings from the Saudi National Mental Health Survey
Source: Front Public Health. 2023 Nov 28;11:1238326. doi: 10.3389/fpubh.2023.1238326 (PMC10715453; doi:10.3389/fpubh.2023.1238326)
Supplement: Supplementary file 1 [file Table_1.docx]

| **Supplementary Table 1. Mental Disorders Interactions of Sociodemographic Correlates and Chronic Physical Conditions** | | | | | | | | | | | | | | |
| --- | --- | --- | --- | --- | --- | --- | --- | --- | --- | --- | --- | --- | --- | --- |
| **Effect** |  | **Any Medical Condition** | | |  | **Any Pain Condition** | | | |  | **Any Physical Condition** | | |  |
|  |  | **Estimate** | **Wald Chi-Square** | **Pr > ChiSq** |  | **Estimate** | **Wald Chi-Square** | | **Pr > ChiSq** |  | **Estimate** | **Wald Chi-Square** | **Pr > ChiSq** |  |
| Any mental disorders * female (ref=male) |  | 0.5271 | 6.4862 | 0.0109 |  | 1.1844 | | 58.1913 | <.0001 |  | 1.1494 | 51.9596 | <.0001 |  |
| Any mental disorders * age 15-22 (ref=50+) |  | -0.9216 | 3.8128 | 0.0509 |  | -0.6951 | | 4.0466 | 0.0443 |  | -0.3555 | 1.0099 | 0.3149 |  |
| Any mental disorders * age 23-30 (ref=50+) |  | -1.1577 | 12.8353 | 0.0003 |  | -0.5092 | | 3.5708 | 0.0588 |  | -0.2359 | 0.7215 | 0.3957 |  |
| Any mental disorders * age 31-49 (ref=50+) |  | -0.2951 | 2.0074 | 0.1565 |  | -0.6114 | | 8.8019 | 0.003 |  | -0.3068 | 2.06 | 0.1512 |  |
| Any mental disorders * high education (ref=low) |  | -0.2713 | 1.2482 | 0.2639 |  | -0.7709 | | 12.8993 | 0.0003 |  | -0.7443 | 11.0768 | 0.0009 |  |
| Any mental disorders * high-average education (ref=low) |  | -0.2997 | 1.6288 | 0.2019 |  | -0.6707 | | 10.2335 | 0.0014 |  | -0.835 | 14.7405 | 0.0001 |  |
| Any mental disorders * low-average education (ref=low) |  | -0.3841 | 2.2639 | 0.1324 |  | -0.9786 | | 18.7579 | <.0001 |  | -1.02 | 18.9501 | <.0001 |  |
| Any mental disorders * high income (ref=low) |  | -0.1206 | 0.4321 | 0.511 |  | 0.1858 | | 1.5303 | 0.2161 |  | 0.0803 | 0.2735 | 0.601 |  |
| Any mental disorders * high-average income (ref=low) |  | -0.5472 | 5.2438 | 0.022 |  | -0.26 | | 2.2407 | 0.1344 |  | -0.4242 | 5.7795 | 0.0162 |  |
| Any mental disorders * low-average income (ref=low) |  | -0.5516 | 3.5469 | 0.0597 |  | -0.3476 | | 3.0537 | 0.0806 |  | -0.645 | 10.3084 | 0.0013 |  |
| Any mental disorders * never married (ref=married) |  | 0.3804 | 2.3623 | 0.1243 |  | -0.1829 | | 0.8743 | 0.3498 |  | -0.2631 | 1.7167 | 0.1901 |  |
| Any mental disorders * separated/divorced/widowed (ref=married) |  | 0.3878 | 2.1508 | 0.1425 |  | 0.1436 | | 0.2053 | 0.6505 |  | 0.4504 | 1.5497 | 0.2132 |  |
| Any mental disorders * rural (ref=urban) |  | -0.2762 | 1.8334 | 0.1757 |  | -0.7978 | | 28.1706 | <.0001 |  | -0.6562 | 18.668 | <.0001 |  |
| Any mental disorders * Central (ref=Western) |  | 0.3637 | 4.5192 | 0.0335 |  | 0.4461 | | 9.9071 | 0.0016 |  | 0.5278 | 13.0703 | 0.0003 |  |
| Any mental disorders * Eastern (ref=Western) |  | 0.1801 | 0.7825 | 0.3764 |  | 0.6775 | | 14.9188 | 0.0001 |  | 0.6581 | 13.3257 | 0.0003 |  |
| Any mental disorders * Northern (ref=Western) |  | -0.4621 | 1.4323 | 0.2314 |  | -0.8194 | | 11.1994 | 0.0008 |  | -0.9442 | 14.7834 | 0.0001 |  |
| Any mental disorders * Southern (ref=Western) |  | -0.0706 | 0.0719 | 0.7886 |  | 0.0923 | | 0.2281 | 0.6329 |  | 0.0596 | 0.0928 | 0.7606 |  |
| Any mental disorders * homemaker (ref=employed) |  | -0.2569 | 1.1079 | 0.2925 |  | -0.2295 | | 1.1458 | 0.2844 |  | -0.3092 | 1.9204 | 0.1658 |  |
| Any mental disorders * other Ψ (ref=employed) |  | -1.3921 | 5.3458 | 0.0208 |  | -1.1775 | | 10.0498 | 0.0015 |  | -1.2499 | 11.3145 | 0.0008 |  |
| Any mental disorders * retired (ref=employed) |  | 0.433 | 2.244 | 0.1341 |  | 0.6508 | | 3.8134 | 0.0508 |  | 0.6406 | 3.4833 | 0.062 |  |
| Any mental disorders * self-employed (ref=employed) |  | -1.2781 | 4.706 | 0.0301 |  | 0.4016 | | 1.7871 | 0.1813 |  | 0.3313 | 1.1731 | 0.2788 |  |
| Any mental disorders * student (ref=employed) |  | -1.115 | 7.3332 | 0.0068 |  | -0.00152 | | 0 | 0.9952 |  | -0.1505 | 0.3437 | 0.5577 |  |
| Any mental disorders * unemployed (ref=employed) |  | -0.6732 | 3.1899 | 0.0741 |  | 0.2488 | | 0.8741 | 0.3498 |  | 0.2099 | 0.5939 | 0.4409 |  |
| Ψ Other includes disabled, maternity leave, sick leave, don't know, refused | | | | | | | | | | |  |  |  |  |
